# Brain microenvironment orchestrates highly aggressive tumor variants: current trends and therapeutic approaches

**DOI:** 10.3389/fnagi.2025.1666837

**Published:** 2025-11-05

**Authors:** Juhi Mishra, John D. Dickinson, Shailendra Kumar Maurya

**Affiliations:** ^1^Department of Biochemistry and Molecular Biology, Massey Cancer Center, Virginia Commonwealth University, Richmond, VA, United States; ^2^Department of Internal Medicine, Division of Pulmonary, Critical Care, and Sleep, University of Nebraska Medical Center, Omaha, NE, United States

**Keywords:** tumor, brain, microenvironment, mortality, therapy

## Abstract

Brain tumors exhibit some of the major challenges in the field of oncology owing to their highly heterogeneous, complex, and aggressive nature. The complex anatomy and aggressiveness of the cancer contribute to high mortality and morbidity worldwide. Moreover, the complexity of genetic mutations and dysregulation molecular processes often culminates into treatment resistance. Consequently, brain tumors have become a serious threat to patients’ lives and overall health. Although advancements in the treatment strategies have been made, but the current knowledge amounts to a drop in the ocean, and many patients still struggling with the disease and exhibit poor prognosis. Hence, there is an urgent need to rigorously expand and fasten the ongoing research to address this clinical challenge. This review explores the components of the brain microenvironment that influence tumor homing and progression toward the aggressive phenotype, with the special emphasis on how these pathways could be therapeutically targeted. The complex milieu of brain niche is further amplified by the infiltrating immune cells, which reshape the brain connectome through novel interactions with resident brain cells. We also discuss the different targeted chemotherapeutic, immunotherapeutic, and combinatorial strategies to limit brain metastasis, which currently has limited therapeutic options. Therefore, this review will discuss all the aspects of brain tumor microenvironment (TME), current strategies, and futuristic insights. We will be discussing the individual components of the tumor microenvironment like BBB, stem cells, astrocytes, immune cells, and non-cellular components like ECM. Further, we also shed some light on current therapies and future strategies targeting these microenvironment components.

## Introduction

The brain microenvironment represents one of the most complex and unique biological territories in the human body, markedly distinct from that of other tumors (Álvaro-Espinosa et al., 2021; Boire et al., 2020). This complexity arises not only from our incomplete understanding of brain homeostasis and the organ’s inherent structural heterogeneity, but also from pathological conditions such as tumors, which further amplify the cellular and molecular diversity of the brain microenvironment (Álvaro-Espinosa et al., 2021). Recent times have seen a rise in the incidence of brain tumors. Although they account for only about 5% of all adult malignancies, however, brain tumors represent up to 70% of solid tumors in children. Additionally, approximately 20%–30% of systemic malignancies eventually metastasize to the brain (Zhao et al., 2017). Both benign and malignant brain tumors can elevate intracranial pressure and compress brain tissue, resulting in CNS dysfunction that may become life-threatening (Zhao et al., 2017). Despite advancements in diagnostic techniques and therapeutic strategies, improvements in overall survival for brain tumor patients remains limited (Zhao et al., 2017). Brain or CNS tumors represent the most prevalent cancer type in individuals aged 0–19 years, where an average annual age-adjusted occurrence rate is 5.42 per 100,000 (Zhao et al., 2017; Gittleman et al., 2014). In adults, the most common types of CNS tumors include meningiomas (15%), glioblastomas (GBs) (20%), and metastatic brain tumors (40%) (Bikfalvi et al., 2023; Tripathy et al., 2024).

**FIGURE 1:**
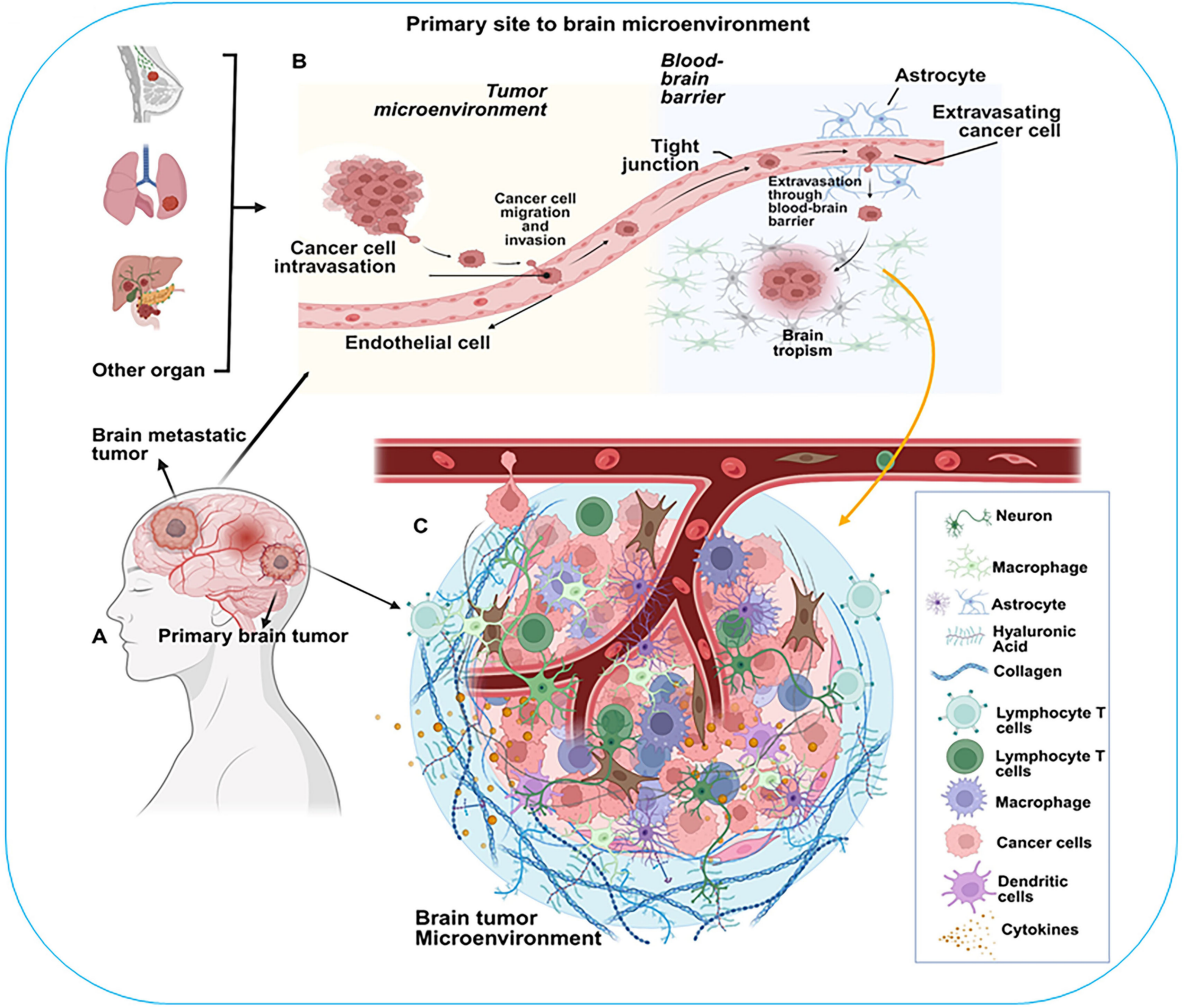
Diagram illustrating the process from primary tumor site to brain microenvironment. **(A)** Brain tumor categorization: primary or metastatica. **(B)** Cancer cell migration from other organs to the brain, highlighting cancer cell invasion, crossing the blood-brain barrier, and astrocyte interaction. **(C)** Brain tumor microenvironment with various cells: neurons, macrophages, astrocytes, lymphocyte T cells, cancer cells, dendritic cells, and cytokines.

The brain TME is a highly diverse structure, both in its timing from early to late disease stages and in its spatial architecture. This variation is noticeable across different tumor types, among individuals with the same diagnosis, between various non-neoplastic cell types and their functional states, and even among individual tumor cell clones (Klemm et al., 2020; Quail and Joyce, 2017; Valiente et al., 2020; Masmudi-Martín et al., 2021; Andersen et al., 2021). All cellular components of the TME, including fibroblasts, pericytes, endothelial cells, glial cells, leukocytes, and tumor cells, engage in complex intercellular communication that promotes brain tumor progression (Figure 1; Quail and Joyce, 2017). A wide variety of immune and stromal cell types, such as dendritic cells (Quail and Joyce, 2017; Pombo Antunes et al., 2021; Yan et al., 2019), neutrophils (Klemm et al., 2020; Zhang L. et al., 2020), macrophages (Klemm et al., 2020; Pyonteck et al., 2013; Bowman et al., 2016; Sankowski et al., 2019; Guldner et al., 2020; Akkari et al., 2020), and astrocytes (Priego et al., 2018; Henrik Heiland et al., 2019), modulate the TME and play crucial roles in shaping T cell responses within brain tumors. In addition to these cellular components, the TME is protected by the blood-brain barrier (BBB), which contributes to the brain’s status as a relatively immune-privileged organ. Immune-privileged organs are characterized by tightly regulated immune activity, leading to an inherently more immunosuppressive environment (Tomaszewski et al., 2019). This unique complexity of the brain underscores the need for comprehensive pharmacological strategies capable of overcoming the specific technical and biological challenges posed by the brain (Álvaro-Espinosa et al., 2021). Recent technological advances have facilitated in-depth multi-omic analyses of the TME, revealing multiple cell subsets and activation states across development, health, and neurodegenerative and neuroinflammatory diseases. In this review, we explored the roles of individual cellular components within the brain TME in driving tumor progression. We also discussed the signaling processes, the mechanisms involved in tumor progression, and their therapeutic significance. A deeper understanding of these cells and related signaling may provide new insights into the development of brain cancers and pave way for the development of more effective therapeutic strategies.

## Brain tumor

Brain tumor is one of the most feared malignancies, with a mortality rate of around 80% (Schwehr and Achanta, 2025). It includes medulloblastoma, one of the common malignant brain tumors in children, and high-grade glioblastoma, the most lethal adult cancers (Louis et al., 2016; Azzarelli et al., 2018). The treatment of these diseases requires both chemotherapy and radiotherapy, which can lead to various adverse effects. Therefore, it is essential to gain a deeper understanding of the biology of these diseases. This knowledge will help ensure that during treatment, only the malignant cells are effectively targeted, sparing the surrounding healthy tissue (Azzarelli et al., 2018).

There are nearly 150 different types of brain cancer, which can be categorized into two main groups: primary and metastatic. The most common tumors in the brain and CNS typically arise from glial cells. Treating these tumors can be challenging due to their diverse growth patterns, and their characteristics are still being explored. It is essential to identify the key features and growth factors of brain tumors, differentiate them from other tumor types, examine treatment options, and investigate drug resistance to improve treatment outcomes. Malignant brain tumors account for the second-highest number of cancer-related deaths in the United States, representing 2.4% of all cancer cases (Sarkar et al., 2023; Azzarelli et al., 2018; Kaza et al., 2012; Xu et al., 2007; Ostrom et al., 2016). Gliomas are the primary brain tumors that originate from the glial cells, and they may be classified as low-grade or high-grade. The low-grade gliomas (grade I and II) are slow growing, usually have better prognosis and may not require aggressive treatment, while high-grade gliomas (grade III and IV) are highly aggressive and require an intense treatment regimen. High-grade gliomas (usually grade IV) are referred as glioblastoma (GB), and are among the most common types of brain (Louis et al., 2007). In addition to glial cells, these tumors may contain nerves, blood vessels, glands, and other cells that contribute to their structure. While most brain tumors that metastasize originate in the brain, some can develop in other areas of the body and spread to the brain through the circulatory system. This is often seen in patients with breast or lung cancers. To better understand their development, outcomes, treatment options, drug resistance, and potential for recurrence, it is crucial to investigate their origins, including the formation of cancer stem and progenitor cells (Sarkar et al., 2023; Azzarelli et al., 2018; Abou-Antoun et al., 2017; Zhang et al., 2025). The classification of brain tumors is based on their type, metastatic potential, and prognosis. The complexity and outlook for brain tumors depend on their origin, development, and progression.

## Brain metastatic tumor

Brain metastasis is a major contributor to intracranial neoplasms and plays a significant role in cancer-related death (Campbell et al., 2022). The probability of cancer spreading to the brain to form a tumor is ten times higher than that of developing primary brain cancer (Campbell et al., 2022). Approximately 8%–10% of cancer patients experience brain metastases, with around 200,000 new cases diagnosed each year in the United States (Vogelbaum et al., 2022; Miccio et al., 2024). Additionally, between 14% and 20% of cancer patients will develop brain metastasis at some point during their treatment (Campbell et al., 2022; Hatiboglu et al., 2013). It shows that every year, 1.7 million new cancer patients are diagnosed in the USA, and around 340,000 are expected to develop brain metastasis during their disease course (Campbell et al., 2022). The occurrence of brain metastases (BrM) at the time of initial cancer diagnosis varies significantly across different cancer types. The highest rates of brain metastases at the time of initial diagnosis are seen in lung cancer and melanoma, with occurrences of 25%. This is followed by renal cancer at 10%, breast cancer at 7%, and head and neck or esophageal cancers at 5%. Non-esophageal metastatic gastrointestinal cancers have an occurrence rate of around 2% (Cagney et al., 2017). Many patients may develop brain metastases after their initial diagnosis. Depending on the type of cancer, the percentage of patients who experience brain metastases within 1 year can vary significantly. For instance, approximately 20% of patients with lung cancer may develop brain metastases, while the rates for patients with breast cancer, renal cell cancer, and melanoma range from 5% to 7% (Vogelbaum et al., 2022; Davis et al., 2012). However, irrespective of the tumor type (primary or metastatic), the surrounding microenvironment influences and guides the tumor progression.

## Brain microenvironment

**FIGURE 2:**
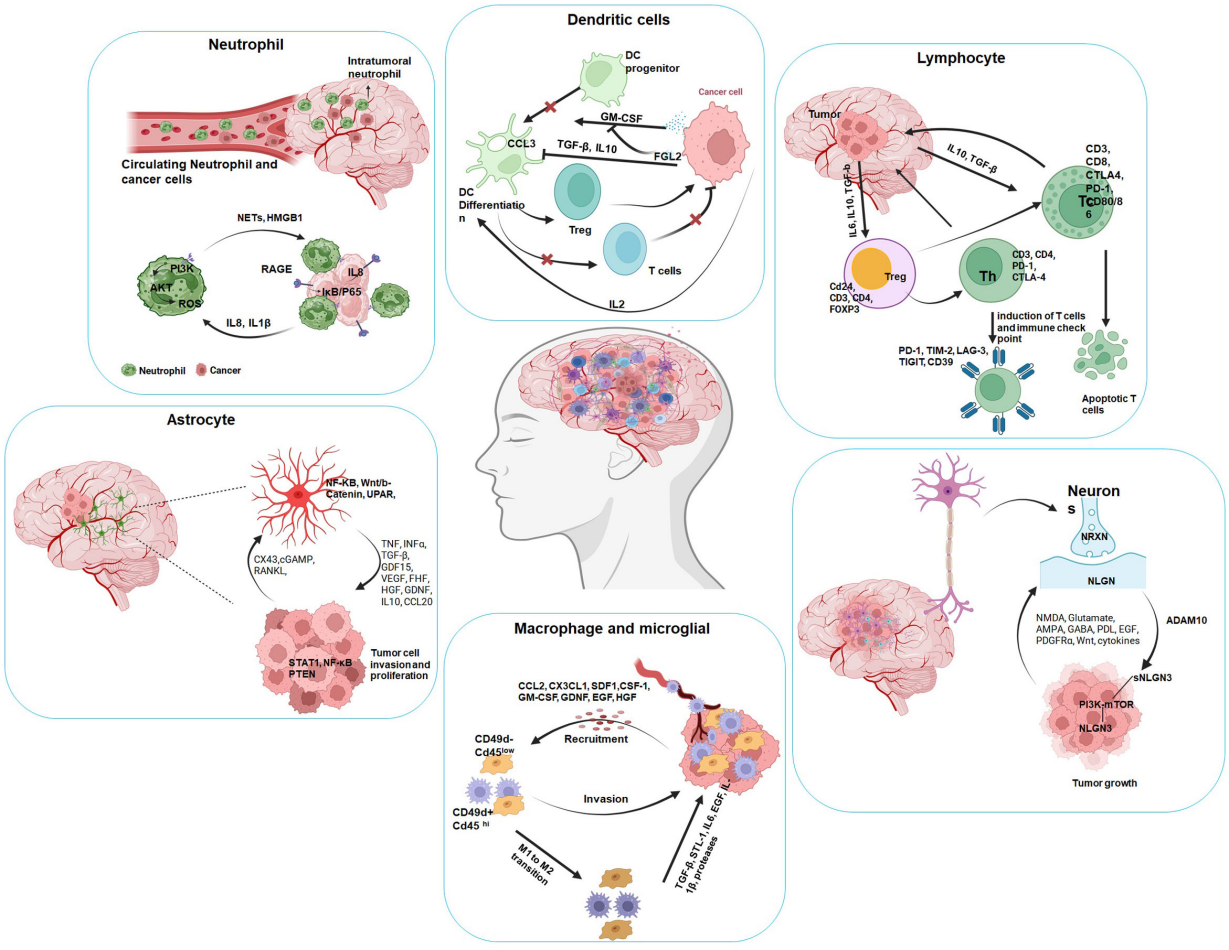
Diagram of brain tumor microenvironment interactions with various cell types. Panels depict neutrophils, astrocytes, lymphocytes, dendritic cells, macrophages, microglia, and neurons. Each panel illustrates cellular pathways and factors like cytokines, signaling molecules, and transcription factors involved in tumor progression, invasion, and immune regulation. Central image shows a brain with highlighted tumor regions. Arrows indicate interactions, signaling pathways, and cellular effects relevant to tumor biology.

The brain TME is a complex and heterogeneous system composed of various components, including cancer cells, different types of brain cells such as neurons, astrocytes, endothelial cells, and oligodendrocytes. It also contains resident immune cells like microglia, tumor-associated macrophages (TAMs), and tumor-infiltrating lymphocytes (TILs). BBB thoroughly regulates the brain microenvironment and keeps it selectively segregated from the systemic blood supply. Therefore, this unique brain feature makes the treatment of the tumors very challenging (Sharma et al., 2023; Martinez-Lage et al., 2019; Plaks et al., 2015). Brain microenvironment also has a role in determining treatment response, thereby influencing tumor progression. Such response is related to a series of interconnected disparities in the spatial cellular organization, the composition of the extracellular matrix, and the cellular landscape (Watson et al., 2024). However, evaluating such a change from a spatial perspective is challenging due to the limitations of current high-dimensional imaging techniques and the level of intratumoral heterogeneity across large lesion areas (Watson et al., 2024). The high-dimensional techniques that have the ability to acquire complex multiparametric biological data that include single-cell RNA-sequencing, time-of-flight mass cytometry, Multiplexed Imaging, Omics Profiling, etc., (Sankowski et al., 2019). The CNS signifies a complex niche that is distinct from the tumor-associated microenvironment (Boire et al., 2020; Álvaro-Espinosa et al., 2021). Additionally, the microenvironment also features some non-cellular components such as exosomes, extracellular matrix (ECM) components, secreted ECM remodeling enzymes, and both autocrine and paracrine signaling molecules. With its diverse composition and disruptive nature, the TME plays a crucial role in the survival and response to therapy of cancer cells (Figure 2; Sharma et al., 2023; Martinez-Lage et al., 2019; Plaks et al., 2015). Therefore, we will be discussing individual components in detail.

## Blood-brain barrier (BBB) and brain tumor microenvironment

The BBB is one of the most densely vascularized structures, made up of tightly connected endothelial cells and surrounded by a basal lamina associated with pericytes and astrocytic foot processes (Lorger, 2012; Pasqualini et al., 2020). This barrier network is poorly connected to the neuron endings and microglia, which can significantly impact the maintenance and regulation of integrity during injury (Pasqualini et al., 2020; Abbott et al., 2006). These vascularized structures act as a selective barrier between the brain’s parenchyma and the circulatory system, playing a crucial role in maintaining brain homeostasis by preventing infections and toxic substances from entering the brain. On the other hand, this positive attribute negatively impacts treatment strategies as it makes the delivery of therapeutics very challenging (Pasqualini et al., 2020; Muldoon et al., 2013).

Malignant brain tumors are among the most vascularized tumors found in humans. In one *in vivo* mouse model experiment, tumor cells growing within the brain exhibited a 50% higher blood vessel density compared to those growing subcutaneously (Lorger, 2012; Blouw et al., 2003; Lorger et al., 2009). This enhanced angiogenesis can be attributed to the increased vascular endothelial growth factor (VEGF) secretion in the brain model as compared to the subcutaneous model (Lorger, 2012; Guo et al., 2001; Deli et al., 2005; Lee et al., 2006; Nag, 2003). Such brain blood vessels are exclusively distinct from those of the other body organs, due to their unique structure and tight junctions. Moreover, the pericyte and astrocyte end feet processes surround the blood vessel, and smooth-muscle cells support blood vessels and contribute to the tightness of the BBB (Deli et al., 2005; Guo et al., 2001; Lee et al., 2006; Nag, 2003). The BBB is altered during the brain cancer progression, either primary or as a metastatic site, resulting in what is termed the brain-tumor-barrier (BTB) (Steeg, 2021). BTB modification alters the barrier permeability and regulatory pathways involving tumor cells (Steeg, 2021). The barrier properties of the BBB are maintained by signals such as Wnt, retinoic acid, and sonic hedgehog, secreted by pericytes and astrocytes and other CNS-resident cells (Liebner et al., 2018; Phatale et al., 2025). The basement membrane, a specialized extracellular matrix secreted by pericytes and brain microvascular endothelial cells (BMECs) is primarily composed of laminins, type IV collagen, nidogens, and heparan sulfate proteoglycans (Phatale et al., 2025). These proteins form an additional barrier, provide mechanical stability, and serve as a signaling platform that mediates vascular homeostasis and communication with surrounding cells (Phatale et al., 2025). Microglia, astrocytes, and pericytes express multidrug resistance protein 1 (MDR1), a key efflux transporter localized on the luminal surface of capillary endothelial cells. MDR1 regulates the passage of molecules from the bloodstream into the brain by actively pumping substrates out, thereby restricting entry of potentially harmful compounds (Phatale et al., 2025; Chai et al., 2022). In addition, MDR1 contributes to the clearance of metabolic waste products from brain cells. Proper expression and function of MDR1 are therefore critical for maintaining brain homeostasis and protecting the central nervous system from toxic insults. However, during the tumor formation, it initiates the leakage of the brain capillaries by increasing the gap in the tight junctions of the BBB, leading to the development of a novel barrier known as the BTB (van Tellingen et al., 2015). The grade of the tumors is directly proportional to the BBB disruption, like in a high-grade carcinoma, BBB disrupts to form a leaky BTB. This transformation could be attributed to the development of the hypoxic environment as a result of the high metabolic activity of the glioma cells. This results in the VEGF overexpression, upregulated angiogenesis, and abnormal blood vessels, culminating in the compromised BTB (Phatale et al., 2025; Yadav et al., 2021). The remodeled architecture of the BTB makes it extremely difficult for small molecules or antibodies to penetrate the tumor site, thereby challenging treatment strategies (Watkins et al., 2014; Arvanitis et al., 2020). Furthermore, similar to BBB, BTB endothelial cells also express drug efflux transporters, which can also impede drug delivery. To make matters worse, ABC transporters in the cancer cells further contribute to drug resistance (Phatale et al., 2025). Notably, the BTB a heterogeneous structure, can impede the entry of therapeutic agents into brain tumors, and addressing this challenge is crucial for enhancing treatment effectiveness and improving the quality of life for patients. Overcoming this challenge is key to improving treatment effectiveness and patient quality of life (Steeg, 2021).

## Astrocyte and brain tumor microenvironment

In the brain TME, astrocytes serve as the primary cells responsible for regulating many essential physiological functions (Zhang H. et al., 2020). In a healthy brain, astrocytes are the most abundant type of cells. These cells provide essential nutrients, support to the neurons, and act as unique stem cells. They possess the remarkable ability to proliferate, adapt to new environments, and form connections with various cellular components. Additionally, astrocytes are responsible for maintaining ionic and neurotransmitter balance, modulating synaptic activity and plasticity, and responding to damage in the CNS (Charles and Holland, 2010; Doetsch, 2003; Massagué and Obenauf, 2016; Quail and Joyce, 2013; Brandao et al., 2019; Zhang H. et al., 2020; Hu et al., 2023). During brain injury, astrocytes become activated and are known as reactive astrocytes (astrogliosis). This activation has both advantageous and detrimental effects on the CNS (Lorger, 2012; Sofroniew, 2005; Sofroniew and Vinters, 2010). The activated astrocyte exhibits increased levels of the protein GFAP (Glial Fibrillary Acidic Protein), which has been significantly elevated near the primary and brain metastatic tumors in animal models and human patients (Lorger, 2012; Nicolson et al., 1996; Lorger and Felding-Habermann, 2010; Fitzgerald et al., 2008; Zhang and Olsson, 1995, 1997). Several *in vitro* experiments indicated that astrocytes released various growth factors that play a crucial role in the growth regulation of both primary and metastatic brain tumor cells. These factors include TGF-α, CXCL12, S1P, and GDNF (Lorger, 2012; Hoelzinger et al., 2007). Notably, the release of IL-6, TGF-β, and IGF-I by astrocytes promotes the proliferation of brain-tropic cancer cells *in vitro* (Lorger, 2012; Sierra et al., 1997). During the lung cancer brain metastasis, lung cancer cells secrete IL-8, MIF, and PAI-1, which activate astrocytes and induce the expression of TNF-α, IL-1β, and IL-6, thereby promoting the proliferation of cancer cells (Lorger, 2012; Seike et al., 2011). In one *in vitro* study, co-culture of lung adenocarcinoma cells with astrocyte cell lines shows activation of ERK1/2 and Akt phosphorylation in cancer cells, enhancing the proliferation by activating these specific signaling pathways (Lorger, 2012; Langley et al., 2009). During the invasion of cancer cells in the brain, astrocyte cells facilitate this process by releasing heparanase, an enzyme that breaks down heparan sulfate proteoglycans in the extracellular matrix (Marchetti et al., 2000; Lorger, 2012). This heparanase expression is upregulated in astrocytes by nerve growth factor (NGF) in response to factors secreted by cancer cells, including TGF-β1, IL-1β, and bFGF (Yoshida and Gage, 1991; Lorger, 2012). Perivascular astrocytes are intricately linked with endothelial cells and play a crucial role in maintaining the integrity of the BBB (Kim et al., 2006; Charles and Holland, 2010). They also enhance the activity of neural stem cells by establishing contact and releasing various diffusible signals (Charles and Holland, 2010; Lim and Alvarez-Buylla, 1999; Song et al., 2002; Környei et al., 2005). In case of glioma, reactive astrocytes exhibit the localized expression of sonic hedge-hog (SHH) and Gli signaling within the perivascular niche, which correlates with the increasing grade of glioma. This SHH/Gli signaling pathway is significant for the self-renewal of brain tumor stem cells (BTSC) and is essential for sustained tumor growth and survival of gliomas (Charles and Holland, 2010; Becher et al., 2008; Clement et al., 2007; Stecca and Ruiz i Altaba, 2005).

During brain metastases, the expression of PTEN, a kind of tumor suppressor gene, is significantly downregulated compared to primary tumors as well as metastases from other common secondary organs (e.g., bone and lung), both in mouse and patient samples (Zhang et al., 2015). A co-culture study reveals that the microRNA released from astrocytes has the potential to suppress PTEN expression within brain metastatic cells, leading to increased activation of PI3K signaling and enhanced cellular outgrowth (Zhang et al., 2015). Inversely, tumor cells secrete RANKL that triggers the astrocytes via NF-κB signaling, thereby increasing tumor-associated astrocytes (TAAs). These activated TAAs release TGF-β and other secretory factors, which promote glioma cell invasion (Kim et al., 2014; Hu et al., 2023). Similarly, glioma cells markedly activate astrocytes by enhancing Wnt/β-catenin signaling, which results in increased degradation of ECM to facilitate tumor invasiveness (Lu et al., 2016; Hu et al., 2023). Factors secreted by TAAs include IL-6, IGF-1, GDF-15, VEGF, FGF, EGF, TNF-α, TGF-β, and HGF, all of which potentially contribute toward increased proliferation (Brandao et al., 2019; Zhang H. et al., 2020; Hu et al., 2023). Additionally, TAA also protects the GBs cells from the hypoxic microenvironment by altering CCL20/CCR6 signaling axis, to promote angiogenesis and enhance tumor cell invasion (Brandao et al., 2019; Hu et al., 2023). Furthermore, they also play a critical role in imparting cancer cells, resistance against radiotherapy and chemotherapy (Doetsch, 2003). On the other hand, TAA-mediated secretion of IL-6, STAT-3, GDF-15, IFN-γ, IL-10, tenascin-C, and PD-L1 protects the GBs cells against immune therapy (Zhang H. et al., 2020; Hu et al., 2023). Overall, alterations in the fundamental TAA-associated signaling could represent a novel approach for GBs treatment.

## Neuron and brain tumor microenvironment

In the brain, neurons serve as the primary cell type and are integral to the underlying tumor progression. It initiates mitogenic signaling in the CNS, thereby promoting the growth of neural stem cells and oligodendroglial precursor cells (Tomaszewski et al., 2019; Liu et al., 2011). A recent study suggests that optogenetic stimulation of neurons enhances the expression of neuroligin-3 (NLGN3), which, in turn, promotes tumor cell proliferation through the PI3K-mTOR tumor-intrinsic pathway in patient-derived xenograft glioma models. Additionally, same study also inversely correlates survival rates and NLGN3 expression in human GBs (Venkatesh et al., 2015). In cases of breast cancer with brain metastasis, heightened expression of neurotransmitters, such as GABA receptors and transporters released by neurons, functions as an oncometabolite (Neman et al., 2014). Moreover, it has now been observed that neuronal activity promotes glioma progression by forming synaptic communications with the cancer cells. This remodeling of synapses results in altered brain circuit activity and tumor growth. Axon guidance cues, especially Semaphorin-4F, facilitate the tumor infiltration and progression to an aggressive phenotype (Huang-Hobbs et al., 2023). This infiltration usually occurs along the white matter fibers where myelinated axons serve as an infiltration cue (Salvalaggio et al., 2023, 2024; Huang-Hobbs et al., 2023). Interestingly, these white matter axonal tract density has now been correlated with the disease prognosis. In a prognostic study performed on 112 patients, it was revealed that a higher axonal tract density is associated with poor prognosis and vice versa. Additionally, this correlation proves to be a strong prognostic marker as compared to the other known markers (Salvalaggio et al., 2023, 2024). Therefore, in the coming times, this prognostic marker will be highly beneficial in understanding disease outcome and planning treatment strategies.

In the brain microenvironment, the interaction between cancer cells, neurons, and glial cells extends beyond the release of various secretory factors. Some studies have indicated that genetic material can also be transferred between these cells through extracellular vesicles (EVs) and cell fusion (Pasqualini et al., 2020). Glioma cells that secrete EVs play a crucial role in regulating key processes involved in tumor progression. They have been shown to facilitate the transportation of signaling molecules, oncogenic genes, receptors, and microRNAs (miRNAs), and directly modulate the TME (Pasqualini et al., 2020; Godlewski et al., 2015; van der Vos et al., 2016). The ability to modulate gene expression in both glial and neuronal cells has been demonstrated through the use of triple transgenic nude mice models, where fluorescently labeled glioma and non-glioma cell types facilitated dynamic glioma development imaging (Pasqualini et al., 2020; Gao et al., 2020). It was observed that glioma cells induce network hyperexcitability to increase neuronal activity and ultimately promote tumor growth (Pasqualini et al., 2020). Additionally, neurons and glioma stem cells (GSC) co-culture study identified the formation of glutamatergic neuron-glioma synapses as one of the mechanisms promoting tumor growth. These synapses enhance glioma growth and invasion by regulating calcium communication within the tumor microtube-connected cell networks (Venkataramani et al., 2019; Pasqualini et al., 2020). Intriguingly, metastatic cancer cells functionally replace astrocytes in some cases of breast-to-brain metastasis, by forming a pseudo-tripartite synaptic framework, to promote tumor cell growth by glutamate release. This glutamatergic signaling activates N-methyl-D-aspartate receptors (NMDARs) on tumor cells, facilitating their colonization and proliferation within the brain microenvironment (Pasqualini et al., 2020; Zeng et al., 2019). Furthermore, non-synaptic, activity-dependent potassium currents are amplified via gap-junction-mediated intercellular connections, establishing an electrically coupled network between neurons and tumor cells. *In vivo* studies have demonstrated that depolarization of glioma cell membranes promotes tumor proliferation, whereas disrupting this electrochemical signaling inhibits tumor growth and significantly improves survival in mouse models (Pasqualini et al., 2020; Venkataramani et al., 2019) thereby providing a novel approach for targeting brain tumors.

## Cancer stem cells and brain tumor microenvironment

Cancer stem cells (CSCs) are a type of self-renewing cell pool that sustains the tumor by regenerating differentiated tumor cells (Kong, 2012). This hypothesis for tumor growth and maintenance has recently received significant attention (Azzarelli et al., 2018; Batlle and Clevers, 2017). In one model, the tumor cells are nourished by a subpopulation of slow-cycling stem cell-like cells that promote the tumor-initiating potential. CSCs are commonly believed to be resistant to therapies and retain the ability to regenerate the diverse cell types within the tumor mass even after treatment concludes. Cancer stem cell-like cells were first identified and isolated from brain tumors in laboratory settings. However, the relationship between this behavior and its function in living organisms is still not entirely understood (Azzarelli et al., 2018; Galli et al., 2004; Singh et al., 2003, 2004). In one study, a CD133+ cell subpopulation isolated from human pediatric brain tumors revealed stem cell-like properties in culture and, during implantation in animals, recapitulated the original tumor’s characteristics, including its heterogeneous cell composition (Singh et al., 2004). The same type of cells with stem-like properties were isolated from the different pediatric tumors, such as glioma, medulloblastoma, primitive neuroectodermal tumors and ependymoma (Galli et al., 2004; Hemmati et al., 2003). Similar to non-malignant neural precursor cells, tumor stem cell-like cells can grow *in vitro*. This allows a comparison between normal stem cells and tumor stem cells, paving the way to identify drugs that specifically target cancer cells without affecting their normal counterparts (Bressan et al., 2017; Pollard et al., 2009).

The interaction between CSCs and various immunosuppressive cells plays a crucial role in the development of the TME and cancer progression (Li et al., 2023; Luo and Yu, 2019; Vahidian et al., 2019). CSCs possess a unique ability to recruit immune cells, including regulatory T cells (Tregs), myeloid-derived suppressor cells (MDSCs), and TAMs to promote immune suppressive environment (Vahidian et al., 2019; Chikamatsu et al., 2011). During tumor progression, CSCs release TGF-β, which promotes further differentiation and enhances the functional characteristics of Tregs (Li et al., 2023). Recruited Tregs secrete vascular endothelial growth factor A (VEGFA), which enhances the stemness and progression of cancer stem cells, while also promoting angiogenesis (Vahidian et al., 2019). Additionally, VEGFA initiates the epithelial-mesenchymal transition (EMT) process in cancer stem cells, thereby increasing their metastatic potential. Within the TME, CSCs stimulate the expansion of MDSCs, creating an immunosuppressive environment. They achieve this by regulating arginase and transforming growth factor-beta (TGF-β), which inhibits T cell infiltration, proliferation, and function (Vahidian et al., 2019; Li et al., 2023). Furthermore, Tissue-associated macrophages (TAMs) play a crucial role in regulating the growth and metastasis of CSCs by secreting various factors such as PDGF, TGF-β, IL-8, and CXCL12, all of which increase the stemness of CSCs (Li et al., 2023). TAMs also secrete milk-fat globule-epidermal growth factor-VIII (MFG-E8), which allows CSCs to boost tumorigenicity and resist anticancer drugs (Li et al., 2023; Jinushi et al., 2011). In different case of cancer, including liver, gastric, colon, and glioma, an increase in the expression of CD90 has been observed in cancer stem cells. This increased expression of CD90 in CSCs has been shown to interact directly with TAMs, further enhancing their stem cell properties (Li et al., 2023).

The brain tumor can originate either from stem, progenitor or more mature cells and the origin of the tumor significantly influences the behavior of the cells involved. Understanding the specific cell types from which each tumor arises can reveal lineage-specific therapeutic vulnerabilities. This knowledge may also help us to identify early malignant or even pre-malignant abnormal cell states, some of which may be more susceptible to oncogenic attacks than others. Although various studies indicate that certain brain tumor subpopulations exhibit stem cell-like behavior, identifying specific cell surface markers for these cells has proven challenging (Azzarelli et al., 2018). Like, cells that are positive for CD133 have been shown to possess tumor-initiating potential. Similarly, cells that are negative for CD133 also exhibit this potential (Beier et al., 2007; Ogden et al., 2008; Read et al., 2009). Additionally, cell surface marker CD15 (stage-specific embryonic antigen, SSEA1) has been suggested as a common marker for brain tumor stem cells for gliomas and medulloblastomas (Son et al., 2009; Ward et al., 2009). In many studies, researchers have identified and isolated glioma stem cells (GSCs) from GBs tumor tissues. These isolated stem cells have the potential to promote tumor angiogenesis by increasing the expression of VEGF (Bao et al., 2006; Hu et al., 2023). Additionally, these cells are closely associated with vascular niches and form networks with endothelial cells, enhancing their self-renewal and tumorigenicity (Thirant et al., 2012). One study by Bao et al. (2006) demonstrated that stem cells isolated from GBs differentiate into pericytes, which support vessel growth and tumor progression in xenograft models (Cheng et al., 2013). These stem cells interact with endothelial cells through the SDF-1/CXCR4 axis and promote vascular pericyte differentiation via TGF-β signaling (Cheng et al., 2013). Furthermore, the selective inhibition of GSCs differentiating into pericytes through HsvTK-induced ganciclovir toxicity disrupts the vascular structure and function of the tumor, ultimately inhibiting GBs growth (Cheng et al., 2013; Hu et al., 2023). Additionally, targeting G-pericytes, the blood-tumor barrier (BTB) hampers and increases BTB permeability by impairing tight junctions, which increases drug delivery to enhance GBs chemotherapy efficacy (Zhou et al., 2017).

## TAMs and microglial cells

Historically, the CNS was thought to have a very limited immune response (Pasqualini et al., 2020; Medawar, 1948; Widner and Brundin, 1988). However, this view has recently been challenged by discoveries such as the presence of functional lymphatic vessels in the meninges, different types of APCs, and the entry of T cells through the BBB. Additionally, it has been shown that immunologically related populations of immune cells, including macrophages, can reside in the meninges (Pasqualini et al., 2020; Absinta et al., 2017; Da Mesquita et al., 2018; Louveau et al., 2015). Alternative routes of cerebral infiltration for immune cells include the meninges and the choroid plexus (Benakis et al., 2018). Based on these observations, it was proposed to refer to the brain as an immunologically distinct rather than “privileged” site.

In the brain’s microenvironment, various subsets of myeloid cells exist. Ontogenetically, there are two main macrophage populations present in the brain TME, namely tissue-resident microglia and bone marrow-derived macrophages (Quail and Joyce, 2017; Lorger, 2012; Davis et al., 1994; Guillemin and Brew, 2004). The perivascular macrophages are the main immune cell population, making up about 30% of the tumor mass. They play a crucial role in immune regulation by presenting antigens at the BBB, with a high turnover rate and regular replacement by blood monocytes (Hickey and Kimura, 1988). In the brain TME, the non-parenchymal macrophages originate from embryonic development and form a largely population of stable cells in adult life (Goldmann et al., 2016). In case of pathological conditions and tissue homeostasis, circulating monocytes are recruited to the brain and differentiate into bone marrow-derived macrophages (BMDMs). In contrast, microglial cells are specialized tissue macrophages that reside in the brain (Streit et al., 2005). Several studies have highlighted the challenges faced by monocytes that infiltrate the adult brain as they undergo differentiation into parenchymal microglia. However, it is important to note that the turnover rate of monocytes in a healthy brain is very low (Lorger, 2012; Davis et al., 1994; Guillemin and Brew, 2004; Cartier et al., 2009; Hess et al., 2004; Lesniak et al., 2005; Priller et al., 2001; Soulas et al., 2009). Microglia generally evolve from embryonic yolk sac progenitor cells (Ginhoux et al., 2010; Gomez Perdiguero et al., 2015) and are not removed by peripheral mononuclear hematopoiesis. Therefore, the microglial cell population in the adult brain is maintained by prolonged cellular longevity and local proliferation.

In the CNS, microglial cells serve as the primary immune effector cells and have the potential to trigger a significant immune response. In a healthy brain, these microglial cells exist in a resting state and are distributed uniformly throughout the brain. Upon the signal induction, these brain-resident resting microglial cells can quickly transform into two distinct morphological states: activated microglia and reactive or amoeboid microglia (Davis et al., 1994; Yang et al., 2010). The active form of microglia have hyperdilated stellate morphology with Class I Major histocompatibility complex (MHCI) expression on their surface. However, the reactive microglia represent amoebal morphology and express both MHCI and MHCII, exhibit increased antigen-presenting capability, along with high phagocytic activity (Kettenmann et al., 2011; Lorger, 2012). The above activation/reactive macrophages and microglial cells that have high expression of F4/80 (mouse) or CD68 (human) are more frequently infiltrating primary and metastatic brain tumors in both mouse models and human patients (Lorger and Felding-Habermann, 2010; Daginakatte and Gutmann, 2007; Fitzgerald et al., 2008; He et al., 2006; Hoelzinger et al., 2007; Roggendorf et al., 1996; Zhang and Olsson, 1995; Lorger, 2012). Both cells represent about 8%–78% of all cells in human gliomas and 4%–70% of cells in human brain metastases (Morantz et al., 1979a,b; Lorger, 2012). These cells can more actively proliferate in the brain TME and rapidly increase their numbers in the surrounding area (Lorger, 2012; Badie et al., 2001; Klein and Roggendorf, 2001). A tracker study with GFP-labeled bone marrow-derived cells revealed an increase in F4/80+ microglia/macrophages, representing newly infiltrating bone marrow-derived monocytes (Lorger, 2012; De Palma et al., 2005; Machein et al., 2003).

In one study, it was reported that microglial neuropilin 1 (NRP-1), a receptor for placental growth factor semaphorin 3A, VEGFA, and tuftsin, could serve as a promising pharmacological target for patients with GBs (Miyauchi et al., 2018; Glinka and Prud’homme, 2008; Gelfand et al., 2014; Nissen et al., 2013; Majed et al., 2006; Andersen et al., 2021). NRP-1-mediated transforming growth factor-β (TGF-β) signaling promotes amplification of the anti-inflammatory genes, thereby restricting glioma-specific immunity (Nissen et al., 2013; Friese et al., 2004; Uhl et al., 2004; Andersen et al., 2021). The administration of EG00229, a selective NRP1 inhibitor, altered gene expression in microglia, enhancing glioma-specific CD8+ T cell immunity and increasing survival in a mouse model of GBs (Miyauchi et al., 2016). Additionally, the increased expression of NRP1 is linked to lower survival rates in patients with GBs). This suggests that inhibiting NRP1, particularly through the use of inhibitors in combination with antibodies targeting the immune checkpoint protein PD-1, may effectively activate T cells that are specific to GBs (Leclerc et al., 2019). A study throws light on the role of glioma-derived factors (GDF), expressing tumor-associated microglia exhibit pro-tumorigenic functions (Vinnakota et al., 2013). These factors (GDF) can induce one of the receptor, toll-like receptor 2 (TLR2) expression in microglia associated with gliomas, supporting tumor progression and invasion (Vinnakota et al., 2013). The glial cell expresses versican, an endogenous TLR2 ligand, which significantly increases the expression of matrix metalloproteinase 14 (MMP14) in microglia that promotes the tumor invasiveness and growth (Hu et al., 2015). Furthermore, TLR9 activation increases the microglial phagocytic machinery as a result of contact between microglia and tumor cells, leading to tumor cell death (Benbenishty et al., 2019). Additionally, in organotypic cultures of glioma, phagocytosis is exhibited following co-activation of TLR3 and TLR9 in microglia (Huang et al., 2020).

During the growth of the tumor in the brain, tumor-generated extracellular membrane particles also play an effective role in modulating the behavior of microglial cells. Recently, research explained that fluorescently labeled extracellular membrane particles produced from mouse glial cells are engulfed by microglia and enhance the functional changes, including the expression of multiple MMP-encoding genes, upregulation of the immune-checkpoint protein PDL1, and the downregulation of pathways involved in tumor sensing such as SIGLEC-H and the G protein-coupled receptor GPR34 (Maas et al., 2020; Kopatz et al., 2013). Additionally, it is found that in human glioma, there is a two-thirds downregulation of the microglial sensome, a receptor that have a role in sensing the local microenvironment (Maas et al., 2020; Hickman et al., 2013). Interestingly, sensome encoding genes are highly express near the tumor core and as well as in the microglia containing the extracellular membrane particles of GBs (Maas et al., 2020; Darmanis et al., 2017). Furthermore, a study on the extracellular membrane particles produced by tumors is essential for identifying additional molecules such as various released protein molecules, microRNAs, and different metabolites. These components may influence microglial responses both within the TME and potentially at distant sites. A study on human BrMs and glioma sequencing data revealed that type I interferon signaling and nuclear factor-κB (NF-κB) signaling are upregulated in BrMs, and contrastingly not in the microglial cells of gliomas. Additionally, microglia associated with BrMs have higher expression levels of CXC-chemokine ligand 8 (CXCL8, also known as IL-8), which is a chemokine known to attract neutrophils (Klemm et al., 2020; Friebel et al., 2020). This sheds light on why BrMs have a more significant infiltration of neutrophils in comparison to that in gliomas. Overall, these results unravel an intricate and differential functional interaction between microglia and tumor cells specific to tumor type.

A variety of secretory products, like cytokines, enzymes, growth factors, and ROS (reactive oxygen species), released by microglia/macrophages, regulate angiogenesis (VEGF), cellular proliferation (e.g., EGF), and invasive properties (e.g., metalloproteases) in primary and metastatic cancer cells within the brain (Davis et al., 1994; Guillemin and Brew, 2004; Fitzgerald et al., 2008; Hoelzinger et al., 2007; Markovic et al., 2005, 2009). Many studies suggest that both microglia and macrophages play a role in tumor progression, such as the inhibition of microglial and macrophage cell activation by using minocycline, which results in decreased proliferation of glioma cells in the Nf1-deficient mouse model (Daginakatte and Gutmann, 2007). In one experimental model, intra-tumoral administration of ganciclovir resulted in 70% decrease in microglia/macrophages in the tumor and an 80% reduction in tumor volume, indicating that microglia/macrophages promote glioma growth (Markovic et al., 2009). Consequently, it suggests that these cells play a significant role in regulating tumor growth, making them a potential target for novel therapeutic strategies.

## Extracellular matrix

The brain ECM constitutes approximately 20%–30% of the total volume and displays unique properties compared with ECMs in other tissues (Lau et al., 2013). The ECM also provides a structural framework for tumor tissues and plays a pivotal role in modulating cellular behavior and signaling pathways within the TME (Wei et al., 2025; Pasupuleti et al., 2024). Continuous remodeling of the ECM influences key processes such as cell migration, proliferation, and differentiation, while also shaping the immune landscape. These dynamic alterations render the ECM a central regulator of tumor invasion and metastasis (Wei et al., 2025; Collado et al., 2024). Interactions between the ECM and tumor cells mediated by integrins, glycoproteins (such as laminin), and proteases (including MMPs) directly influence tumor biology and contribute to the progression of tumors toward malignancy (Wei et al., 2025; Yuan et al., 2023). Moreover, the heterogeneity of the ECM is closely linked to therapeutic resistance, immune suppression, and the EMT (Wei et al., 2025). Unlike the peripheral Brain ECM is enriched in proteoglycans, glycoproteins, and glycosaminoglycans, especially heparan sulfate proteoglycans (HSPGs) and hyaluronic acid (HA), while deficient in fibrous proteins such as collagens and fibronectins (Day et al., 2025). Within the brain parenchyma, chondroitin sulfate proteoglycans (CSPGs) and heparan sulfate proteoglycans (HSPGs) predominate, serving critical functions in neuronal development, cell signaling, and tumor progression (Day et al., 2025).

Due to its unique composition and properties, the ECM plays a crucial role in regulating tumor cell niches, invasion, and angiogenesis processes that differ from those in other tissues and organs (Quail and Joyce, 2017). Multiple signaling molecules, including chemokines (chemoattractant protein families), interleukins, EGF, TGF, and tenascin, are upregulated and play crucial roles in stimulating signal transduction pathways that drive malignant tumor growth through their respective receptors (Zhao et al., 2017). However, comprehensive analyses of the ECM in various brain tumors are still scarce, impeding our understanding of ECM regulated tumorigenicity. In glioma, interleukins, EGF, fibronectin, and HSPG are frequently overexpressed (Zhao et al., 2017) and positively regulate the cell adhesion, proliferation, growth, metastasis, and wound healing processes, thereby contributing to glioma progression and TME remodeling (Zhao et al., 2017; Quail and Joyce, 2017). These macromolecules act as reservoirs for heparin-binding angiogenic growth factors, such as fibroblast growth factors (FGFs) and VEGFs, which are locally released through the activity of heparanase (Kundu et al., 2016). Moreover, vessel-associated macromolecules such as tenascin C (TNC) and periostin are also upregulated (Brösicke and Faissner, 2015; Mustafa et al., 2012) and promote cancer cell survival (Oskarsson et al., 2014). Additionally, periostin can be secreted by glioma stem cells, facilitating the recruitment of tumor-promoting M2-like macrophage progenitors from the peripheral circulation (Zhou et al., 2015), leading to suppressed immune response. On the other hand, stroma- and ECM-regulated mechanisms can physically block T cells across different tumor types, posing a major challenge to the delivery and effectiveness of immunotherapies (Joyce and Fearon, 2015). This barrier presumably contributes to the immune suppression. For instance, elevated concentrations of TNC in glioma-associated blood vessels seem to “trap” T cells and prevent their migration into the brain tissue (Huang et al., 2010; Quail and Joyce, 2017). Physical properties of the ECM also play a critical role in glioma biology, where a study states that brain ECM stiffness positively correlates with tumor grade (Quail and Joyce, 2017). This increased stiffness was linked to higher levels of TNC and HA, regulated in a HIF1α-dependent way. Importantly, the mutational status of glioma cells affected ECM stiffness; for example, mutations in the metabolic regulator isocitrate dehydrogenase 1 (IDH1) correlate with lower TNC expression, ECM stiffness, and mechanosignaling, thereby improving patient prognosis (Quail and Joyce, 2017). Therefore, a deeper analysis of genetic mutations and their effects on other components of the TME in gliomas and other brain cancers is an urgent need to pave the way for novel therapeutic targets and personalized medicine.

## Dendritic cells

Dendritic cells (DCs) are a type of myeloid cell that function as highly potent APCs, inducing T cell responses through both innate and adaptive immune mechanisms (Hu et al., 2023; Brandao et al., 2019; Louveau et al., 2015). DCs in the brain can be categorized into two subpopulations: myeloid dendritic cells (mDCs) and plasmacytoid dendritic cells (pDCs). In glioma, pDCs contribute to tumor progression in mouse models. In contrast, the elimination of pDCs increases the survival time of the mice by reducing the Tregs number and their suppressive function (Tregs) (Dey et al., 2015). Glioma cells impair the normal functioning of DCs by increasing the secretion of TGF-β and IL-10. Additionally, FGL2 secreted by the glioma cells which hinders with the development of DCs by blocking GM-CSF. This process occurs due to the repression of NF-κB, STAT1/5, and p38 activation. As a result, there is no activation of CD8+ T cells, contributing to the progression of GBs (Yan et al., 2019). Initially, it was exemplified that microglia are one of the primary APCs in the brain, while DCs play a less significant role (Hart and Fabre, 1981; Hickey and Kimura, 1988; Lowe et al., 1989; Ulvestad et al., 1994; Quail and Joyce, 2017). To advance cancer therapy research toward identifying potential therapeutics, one of the best strategies is to utilize the potential immune checkpoint inhibitors. Additionally, the significant clinical benefits of DC vaccines have emerged as another option for stimulating T cell responses (Anguille et al., 2014; Palucka and Banchereau, 2012). As revealed by the clinical trial data from the DC vaccine DCVax-L, better patient survival has been observed as compared to radiation and temozolomide chemotherapy (Stupp et al., 2005). On this basis, a Phase III trial has now been initiated (ClinicalTrials.gov identifier: NCT00045968), highlighting its importance for therapeutic purposes.

### Neutrophils

Neutrophils are among the most potent blood cells, comprising approximately 50%–70% of all circulating leukocytes, and play a significant role in tumor growth and progression. In primary brain tumors, including gliomas, a high level of neutrophil infiltration is commonly observed.

Fossati et al. (1999) and Hu et al. (2023) a higher infiltration correlates with the glioma progression and patient outcomes has been identified as an important prognostic factor. A report from Wang et al. (2020) revealed that glioma patients with poor prognosis displayed elevated levels of neutrophils and also an increased neutrophil-to-lymphocyte ratio (NLR) (Hu et al., 2023). These tumor-infiltrating neutrophils (TINs) release substantial amounts of neutrophil extracellular traps (NETs), which promote aggressive tumor cell proliferation and invasion. This suggests that NETs may serve as an oncogenic marker of high-grade gliomas (HGGs) (Zha et al., 2020). Additionally, the increased number of neutrophils works as a prognostic indicator in IDH wild-type GBs patients treated with the chemotherapeutic drug temozolomide (Wang et al., 2020). During anti-VEGF therapy, an increase in tumor-infiltrating neutrophils was observed, which contributed to resistance to treatment and facilitated tumor progression. Concurrently, the expression of S100A4 was upregulated, promoting glioma cell proliferation and migration (Liang et al., 2014). Therefore, the drug that targets the S100A4 and neutrophils, together with anti-angiogenic therapies, could be a good strategy to slow glioma growth and reduce treatment resistance. Growing evidence highlights the mechanisms underlying neutrophil recruitment in the glioma microenvironment. Notably, in GBs, tumor cells that ectopically express high levels of CD133 enhance neutrophil recruitment via the interleukin-1 (IL-1) signaling pathway, both *in vitro* and *in vivo*. This suggests that CD133-positive tumor-initiating cells may shape a distinct TME through co-evolution with infiltrating neutrophils (Lee et al., 2017). IL8, another potent cytokine acting as a chemoattractant, promotes neutrophil infiltration in the tumor and enhances tumor cell proliferation (Zha et al., 2020). As mentioned earlier, TINs are associated with the formation of NETs, and they also contribute to the production of high-mobility group box 1 (HMGB1) by utilizing PI3K/AKT/ROS signaling axis. HMGB1, a key component of NETs, binds to the receptor for advanced glycation end products (RAGE) on tumor cells, thereby activating the NF-κB signaling pathway. This activation stimulates interleukin-8 (IL-8) secretion, which further facilitates glioma progression (Zha et al., 2020). Importantly, neutrophils possess an intrinsic ability to cross the BBB and the blood–brain tumor barrier (BBTB), enabling their infiltration into glioma tissue. Surgical resection of gliomas further contributes to an inflammatory microenvironment by releasing cytokines such as IL-8, which enhances neutrophil activation and recruitment to the tumor site. The natural tendency of neutrophils to target tumor cells and their high responsiveness to inflammatory signals make them a good candidate for drug delivery systems, where they could prove to be a promising therapeutic strategy for glioma treatment with enhanced specificity and efficacy (Müller et al., 2015).

### Lymphocytes

The lymphoid lineage encompasses key immune cells, including cytotoxic (CD8^+^), helper (CD4^+^), and regulatory (FoxP3^+^) T cells, as well as B cells and natural killer (NK) cells (Hermelo et al., 2025). Among these, CD8^+^ T cells are particularly vital for tumor cell clearance, and their infiltration alongside CD3^+^ T cells into the TME is associated with improved patient survival in glioma (Hermelo et al., 2025; Kmiecik et al., 2013). However, as tumor progress, tumor cells adopt various mechanisms to evade T cell-mediated antitumor responses. For example, glioma cells secrete immunosuppressive cytokines such as TGF-β and IL-10, which inhibit immune activation and suppress the expression of MHC class II molecules on monocytes (Perng and Lim, 2015). Furthermore, IL-10 promotes the upregulation of PD-L1 on monocytes and TAMs, leading to the suppression of lymphocyte activity. Elevated PD-L1 expression is strongly correlated with poor prognosis in glioma patients (Nduom et al., 2016; Bloch et al., 2013). Generally, the naïve CD4+ T cells get transformed into different subclasses of T cell types, including Th1, Th2, Th9, Th17, and Tregs, each with distinct immunological roles (Noor et al., 2024; Yang et al., 2020). These CD4^+^ T lymphocytes are pivotal in orchestrating anti-tumor immune responses in humans. They not only enhance tumor suppression by activating cytotoxic CD8^+^ T cells but can also directly contribute to tumor eradication through certain effector subsets (Yang et al., 2020; Noor et al., 2024; Sacher et al., 2020). Although cytotoxic CD8^+^ T lymphocytes are essential for tumor cell elimination, they may suppress CD4^+^ T cell functions and often lack robust effector-memory capabilities. Moreover, they are prone to exhaustion within the TME. CD8^+^ T cell function is sustained by CD4^+^ T cells, which promote their activation, maturation, and differentiation into effector-memory cells (Noor et al., 2024; Joyce and Fearon, 2015). CTLA-4 is an immune checkpoint receptor that negatively regulates T cell activation and function. Its expression is upregulated in aggressive cancers and is modulated by low levels of the co-stimulatory ligands CD80/CD86 (Liu et al., 2020). In glioma, the number of circulating T cells is reduced to approximately one-third of that in healthy individuals, largely due to impaired egress from the bone marrow. This phenomenon is associated with internalization of the sphingosine-1-phosphate receptor 1 (S1P1); inhibition of S1P1 internalization has been shown to restore T cell release from the bone marrow (Chongsathidkiet et al., 2018). This reduction in peripheral T cell numbers contributes to the classification of gliomas as “cold tumors,” characterized by low immune cell infiltration. Among the immunosuppressive cell types, CD4^+^CD25^+^FoxP3^+^ Tregs are particularly pro-tumorigenic due to their potent immunosuppressive functions across various cancers. Tumor-derived antigens from dying and proliferating tumor cells promote the recruitment of Tregs to the TME. Additionally, tumor or DC derived TGF-β enhances Treg enrichment. Chemokines such as CCL22 and CCL2 secreted by GBs cells further facilitate Treg trafficking to tumor sites (Chang et al., 2016; Crane et al., 2012).

## Therapeutic approaches to target the brain tumor microenvironment

Numerous preclinical and clinical strategies have been developed to explore targeted treatments related to the brain TME, including surgical resection, chemotherapy, and radiation therapy (Quail and Joyce, 2017). Usually, benign or easily accessible tumors are surgically removed and have shown improved survival outcomes. Chemotherapy has shown favorable responses in some cases; however, its efficacy is significantly limited by the presence of the BBB (Quail and Joyce, 2017). Therefore, the primary target of the therapeutic strategy is to target BBB permeability, followed by finding and delivering suitable therapeutic drugs. The treatment strategies discussed below are categorized as BBB targeted, cellular-component targeted, cellular-pathways targeted (angiogenesis and chemokines), followed by immunotherapies.

The BBB restricts the entry of many chemotherapeutic agents, which must traverse the vascular endothelium to reach tumor cells a process highly dependent on the lipophilicity of the drug. As a result, the therapeutic potential and effectiveness of many chemotherapeutic agents are substantially diminished due to limited permeability across the BBB (Zhao et al., 2017). Several preclinical and clinical studies are currently underway targeting the brain TME. A lot of research is now targeted toward enhancing the BTB permeability for efficient drug delivery. Recent strategies are utilizing focused ultrasound-guided (FUS) technique to open up the barrier and deliver drugs. Here, microbubbles are intravenously injected and then, in response to the ultrasounds, they oscillate, creating shear stress in the endothelial cells. This mechanical stress ultimately ruptures the tight junctions, thereby enhancing drug delivery efficiency (Zhang et al., 2023; Mungur et al., 2022). On the other hand, efforts are being made to employ nanoparticle-mediated drug delivery to improve drug uptake. The drug-loaded nanoparticles are transported across the barrier by receptor-mediated transcytosis or shutter peptide-mediated mechanisms. These strategies are proving to be promising; however, they are still in an early stage. Therefore, extensive research and clinical trial studies are needed (Zhang et al., 2023; Liu et al., 2022).

Cancer stem cells are one of the major drivers of tumor progression and recurrence. Therefore, targeting CSCs is a critical strategy for eliminating brain tumors (Charles and Holland, 2010). Brain tumor stem cells (BTSCs), which typically reside in the perivascular niche (PVN) of the brain, rely on several intracellular pathways to maintain their self-renewal, proliferation, and migration. Among these, the Sonic Hedgehog (SHH), PI3K/AKT, Notch, and nitric oxide (NO)/cGMP signaling pathways are particularly important (Charles and Holland, 2010). Inhibitors targeting these pathways have shown promise in suppressing glioma progression and enhancing the responsiveness of brain tumors to therapy (Charles and Holland, 2010; Hambardzumyan et al., 2008; Bar et al., 2007; Fan et al., 2006; Momota et al., 2005; Wachsberger et al., 2005). Additionally, other signaling cascades, including the DNA damage checkpoint kinases Chk1 and Chk2, the Wnt pathway, and the BMP/Smad axis, are also involved in regulating brain tumor development. Targeting these pathways with specific inhibitors has demonstrated efficacy in halting tumor growth in both experimental and preclinical models (Piccirillo et al., 2006; Wurdak et al., 2010; Bao et al., 2006). While targeted therapies used alone or in combination have shown substantial success in improving outcomes for patients with primary tumors, there remain limited options for treating brain metastases (Gautam et al., 2020; Maurya et al., 2025). Notably, in animal studies, the use of targeted agents alone, such as PLB1001, and/or in combination, such as neratinib and cabozantinib, has significantly inhibited both primary tumor growth and brain metastatic lesions (Gautam et al., 2020; Maurya et al., 2025). A variety of therapeutic approaches targeting epigenetic alterations are currently under investigation, offering promising avenues for treating BrM, which are significantly influenced by such modifications. These therapies aim to reverse aberrant patterns of DNA methylation, histone acetylation, and other chromatin modifications that drive tumor initiation and progression. Currently some of these modulators are already in clinical trials, while some already got FDA approved (Maurya et al., 2024). Neutrophils are also emerging as potential prognostic markers in both primary brain tumors (Bertaut et al., 2016; Fossati et al., 1999) and metastatic brain disease (Koh et al., 2016; Mitsuya et al., 2017; Serdarevic et al., 2016). Astrocytes are another therapeutic target under investigation.

The anti-angiogenic monoclonal antibody targeting VEGFR-2, DC101, significantly suppressed malignant glioma growth in experimental models (Kunkel et al., 2001). Treated tumor-bearing mice exhibited reduced tumor volumes and microvessel density compared to controls, which correlated with decreased tumor cell proliferation and increased apoptosis. However, DC101 monotherapy was associated with enhanced tumor invasiveness a phenomenon that was mitigated when combined with EGFR inhibition (Lamszus et al., 2005). Similarly, PTK787, a tyrosine kinase inhibitor targeting both VEGFR and PDGFR, also led to marked reductions in tumor volume and vascular density (Charles and Holland, 2010). The expression of VEGF is high in both Primary and metastatic brain tumors and is responsible for the high vascularization (Jain et al., 2007). This high expression of VEGF gives us a thought to target the brain tumor growth with anti-angiogenic therapies. In one of the phase II clinical trials, administration of cediranib, a pan-VEGF receptor tyrosine kinase inhibitor, promotes a rapid and prolonged vascular normalization in GBs patients. This promotes the increase in vasogenic edema that normally results from an increase in intracranial pressure, a reason for morbidity in brain tumors patients (Batchelor et al., 2007). A similar kind of observation comes out like normalization of tumor blood vessels, and a decreased tumor blood volume results in the prolongation of the survival in an animal model of brain metastasis and glioma patients treated with cediranib or bevacizumab, an anti-VEGF antibody (Lorger, 2012). However, anti-angiogenic therapies have frequently been shown to elevate the continuous progression of primary and metastatic brain tumors in experimental models. This occurs through the integration of precursory blood vessels in the nearby healthy brain parenchyma, ultimately leading to increased cancer cell invasion (Lorger, 2012; Kienast et al., 2010; Du et al., 2008; Pàez-Ribes et al., 2009). Also, bevacizumab does not affect melanoma cells that usually grow in experimental brain metastasis models (Kienast et al., 2010). Further, Clinical trials are evaluating the endothelin receptor antagonist macitentan (NCT01499251) and the cyclooxygenase inhibitor meclofenamate, which modulates gap junctions (NCT02429570). These agents have shown encouraging preclinical outcomes in both primary brain tumors (Kim et al., 2015)(Kim et al., 2015) and brain metastases (Chen et al., 2016; Lee et al., 2016). Chemokine expression modulation is a key contributor to tumor growth and the organotropic spread of metastatic cells. Altered chemokine profiles can modulate cancer cell activation, proliferation, and migration under pathological conditions. As such, targeting dysregulated chemokines and their receptors has become an attractive therapeutic strategy for both primary brain tumors and BrM. A number of immunotherapeutic, chemotherapeutic, and combinatorial approaches focusing on chemokine signaling are currently being evaluated in preclinical and clinical studies (Maurya et al., 2022).

Immune checkpoint inhibitors are also gaining increasing attention for the treatment of both primary and metastatic brain tumors. For instance, in primary brain tumors, nivolumab is being tested with radiotherapy in newly diagnosed GBs (NCT02617589, Phase III), and nivolumab and/or ipilimumab are being compared with bevacizumab in recurrent GBs (NCT02017717, Phase III) (Preusser et al., 2015). Ipilimumab in combination with either nivolumab or fotemustine (NCT02460068, Phase III) (Berghoff et al., 2016) is currently being studied for brain metastatic patients. In parallel, adaptive T cell therapies such as IL13Rα2-targeted chimeric antigen receptor (CAR) T cells are also gaining attention owing to their effectiveness against tumor growth in glioma and other tumor models. Notably, a positive correlation has been observed between higher IL13Rα2 expression tumor grade and it serves as a prognostic marker associated with poor patient survival (Chantrain et al., 2006). Personalized cancer immunotherapies have recently emerged as compelling alternatives to conventional treatments. Among these, cytokine-induced killer (CIK) cells represent a potent therapeutic strategy. CIK cells are MHC-unrestricted cytotoxic lymphocytes generated *in vitro* from peripheral blood mononuclear cells (PBMCs) via stimulation with interferon-gamma (IFN-γ), interleukin-2 (IL-2), and anti-CD3 monoclonal antibodies (Brown et al., 2013).

Macrophages can also be targeted, and one such approach involves targeting TAMs using CSF-1R inhibitors in GBs patients, either in the recurrent setting or in combination with standard-of-care treatments. For example, PRD001 (the anti-PD-1 agent) and BLZ945 combined in solid tumors, and recurrent GBs (NCT02829723, Phase I/II), and for newly diagnosed GBs patients, PLX3397 is being tested with temozolomide and radiotherapy (NCT01790503, Phase Ib/II) (Quail and Joyce, 2017; Butowski et al., 2016). DCVax-L, a DC vaccine, has shown promising results and has advanced into Phase III clinical trials for newly diagnosed GBs (NCT00045968) (Prins et al., 2011). Recently, researchers have utilized the idea of deploying cells of the TME as cellular vehicles for the targeted delivery of therapeutic agents (Lorger, 2012). A research group utilized genetically modified TAMs that were engineered to express interferon-alpha (IFN-α) to target cancer cells. These TEMs were transplanted into the brain tumor, enabling the localized delivery of IFN-α. Natural homing ability of TEMs to the tumor site leads to the significant upregulation of IFN-inducible genes, which in turn, is responsible for the reduced angiogenesis and vascular normalization. Thereby, leading to the tumor suppression with no systemic toxicity (Lorger, 2012; De Palma et al., 2008).

## Conclusion

Several studies over the past few decades have demonstrated that the TME is a key regulator of cancer growth, progression, and therapeutic response in both primary and metastatic brain tumors. The brain TME is composed of a heterogeneous population of cells, including cancer cells, astrocytes, neurons, various immune cells, and TAMs /microglia. All the components of the brain TME collectively influence tumor dynamics, sometimes promoting tumor growth and therapy resistance, while in other cases, suppressing tumor initiation and progression. Several ongoing clinical trials targeting the key signaling molecules involved in these processes are proving to be promising. Moreover, some findings on CSF-1R inhibition and anti-PD-1 agents such as PRD001 have been demonstrated to be effective in brain tumor patients. However, despite these advancements, significant challenges persist. A deeper understanding of the complexity of the brain TME, including the diverse secretory molecules released by various cell types is essential for developing more effective therapeutic strategies aimed at targeting or reprogramming the TME. It is increasingly necessary to move beyond isolated analyses and adopt a more integrative approach that encompasses all cellular and non-cellular components of the TME. Such comprehensive insights are likely to emerge from detailed comparative studies examining how different molecular subtypes of brain tumors shape their surrounding microenvironment during cancer progression. Although it is well-established that molecular subtypes of brain tumors exhibit distinct evolutionary patterns and therapeutic responses, a systematic dissection of TME determinants remains in its infancy and is largely underexplored in clinical settings. Furthermore, it will be crucial to thoroughly investigate how both standard-of-care treatments and emerging investigational therapies affect all aspects of the TME across various brain tumor types and their molecular subtypes. Therefore, a deep and thorough understanding will eventually unlock the doors to more sophisticated and effective treatment designs.
